# Preparation of LuAG Powders with Single Phase and Good Dispersion for Transparent Ceramics Using Co-Precipitation Method

**DOI:** 10.3390/ma8085247

**Published:** 2015-08-19

**Authors:** Liangjie Pan, Benxue Jiang, Jintai Fan, Qiuhong Yang, Chunlin Zhou, Pande Zhang, Xiaojian Mao, Long Zhang

**Affiliations:** 1 School of Materials Science and Engineering, Shanghai University, Shanghai 200444, China; E-Mails: panliangjie@shu.edu.cn (L.P.); yangqiuhong@shu.edu.cn (Q.Y.); 2 Shanghai Institute of Optics and Fine Mechanics, Chinese Academy of Sciences, Shanghai 201800, China; E-Mails: zhouchunlin1989@yeah.net (C.Z.); zhangpande2015@foxmail.com (P.Z.); xmao@siom.ac.cn (X.M.)

**Keywords:** co-precipitation method, single phase, pH values, LuAG powder, transparent ceramics

## Abstract

The synthesis of pure and well dispersed lutetium aluminum garnet (LuAG) powder is crucial and important for the preparation of LuAG transparent ceramics. In this paper, high purity and well dispersed LuAG powders have been synthesized via co-precipitation method with lutetium nitrate and aluminum nitrate as raw materials. Ammonium hydrogen carbonate (AHC) was used as the precipitant. The influence of aging time, pH value, and dripping speed on the prepared LuAG powders were investigated. It showed that long aging duration (>15 h) with high terminal pH value (>7.80) resulted in segregation of rhombus Lu precipitate and Al precipitate. By decreasing the initial pH value or accelerating the dripping speed, rhombus Lu precipitate was eliminated and pure LuAG nano powders were synthesized. High quality LuAG transparent ceramics with transmission >75% at 1064 nm were fabricated using these well dispersed nano LuAG powders.

## 1. Introduction

Laser pulses with high repetition rate and high peak power have very wide applications, for example, extreme ultra-violet light (EUV) source and industry [1]. In order to realize high pulse and high repetition rate simultaneously, many gain materials such as APG glass, Yb:S-FAP, Yb:YAG, *et al.* [2,3,4,5,6] have been studied in detail. But unfortunately almost no one can completely meet the requirements.

Recently, transparent polycrystalline ceramic laser materials have attracted much attention. Because the optical quality of ceramic has been greatly improved, and whose efficiencies are superior or comparable to some of single crystals for highly efficient laser oscillations [7,8]. Nd:LuAG ceramic [9] is attractive and popular recently because of its high thermal conductivity (9.6 W·m^−1^·K^−1^), suitable emission cross-section (9.7 × 10^−20^ cm^2^), long life time (304 μs) and easy to get large scale. To achieve high quality transparent polycrystalline LuAG, the synthesis of pure and well dispersed lutetium aluminum garnet (LuAG) powder is crucial and important. Co-precipitation method using ammonium hydrogen carbonate (AHC) as the precipitant is a relatively simple way to synthesize powder with excellent dispersion and sintering properties [10]. And it has many advantages such as atomic level mixing of high-purity precursors, low processing temperature, low cost, and mass-produced synthesis route [11,12]. In the co-precipitation process, parameters during the synthesis process of YAG powder are of important reference to LuAG powder, such as reaction temperature [13,14], dripping speed [15], aging time [16,17], pH value [16,18], species and dosage of precipitant [11], species and dosage of dispersant [19,20], *etc*. Because most of these parameters are all interdependent during the formation and evolution of the precursor, they need to be optimized carefully. However, the synthesis of LuAG powder using AHC as the precipitant is rarely studied in detail, especially for the influences of pH values, aging time, dropping speed, and the amount of solution.

In this paper, the LuAG powders with single phase and good dispersion were synthesized via co-precipitation method using AHC as the precipitant. The effect of aging time, pH values, and dropping speed on the morphology and phase evolution of the LuAG precursors were investigated. The mechanism of Lu precipitate forming flakes is also preliminarily discussed. The optical properties and microstructures of the resultant LuAG ceramics were discussed systematically.

## 2. Results and Discussion

**Figure 1:**
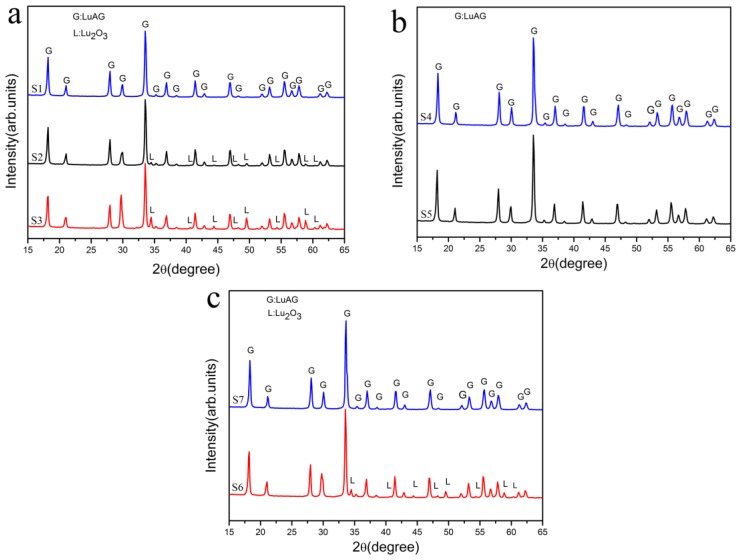
XRD patterns of the powders of S1–S3 (**a**); S4 and S5 (**b**); S6 and S7 (**c**) calcined at 1200 °C for 3 h.

Titration conditions such as aging time, dripping speed, and related pH values of solutions are listed in Table 1. XRD patterns of samples S1, S2, and S3 with different aging time calcined at 1200 °C for 3 h are shown in Figure 1a. It can be seen that no impurity phase except LuAG phase (JCPDS card No. 73-1368) was observed in the powder calcined from1 hour-aged sample S1. As aging time increased to 15 h, a tiny peak of Lu_2_O_3_ (JCPDS card No. 86-2475) phases appeared in the calcined S2 (24 h). When the aging time was prolonged to 24 h (S3), diffraction peaks of Lu_2_O_3_ increased remarkably. We can deduce that the Lu_2_O_3_ impurity phases increased as the aging time was extended.

**Figure 2:**
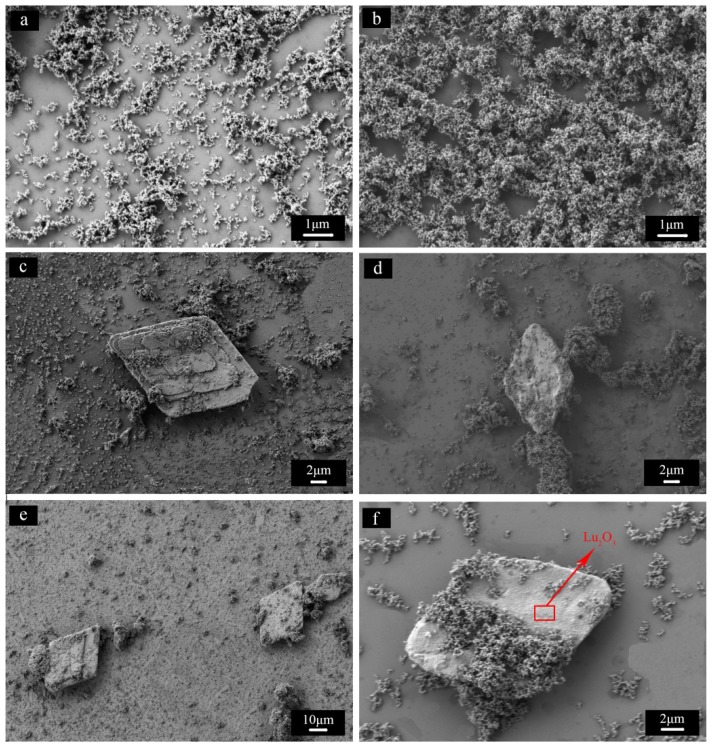
SEM images of the precursors and the resultant powders calcined at 1200 °C for 3 h of S1 (**a**,**b**), S2 (**c**,**d**), and S3 (**e**,**f**).

Figure 2 shows the effect of aging time on the SEM morphology of the resultant precursors and their calcined powders. In order to give a typical view, low magnifications were applied. It can be seen that in the precursor of S1 and its calcined powder, only nano-particles were observed. As the aging time increased, flakes appeared in the precursors and the resultant powders of samples S2 and S3 calcined at 1200 °C for 3 h. Energy-dispersive X-ray spectroscopy (EDS) of the selected area of the flake in sample S3 (Figure 2f) shows that the flake is Lu_2_O_3_, which is consistent with the XRD results. The appearance of flakes in the aged precursors indicates that the flakes were not from titration process or calcining process but a result of segregation of Lu precipitate during aging.

**Table 1:** Titration condition of different samples.

Samples	Aging Time	Dripping Speed	pH Value of Initial Salt Solution	pH Value of Terminal Solution
S1	1 h	3 mL·min^−1^	3.10	7.77
S2	15 h	3 mL·min^−1^	3.10	7.86
S3	24 h	3 mL·min^−1^	3.10	8.04
S4 *	0.5 h	3 mL·min^−1^	3.10	8.35
S5	24 h	3 mL·min^−1^	1.20	7.32
S6	1 h	1.5 mL·min^−1^	3.10	7.90
S7	15 h	6 mL·min^−1^	3.10	7.61

* The AHC solution for preparation of S4 was stirred 4 h and the pH value of which increased to 8.83 before dripping.

Aging is considered important for the preparation of the YAG or LuAG precursor in the precipitation process [17,21,22], because it is believed that ions and molecules in the precipitate rearranged to form better crystallized compound, which helps the washing out of byproducts. The formation of ammonium salt was also enhanced during aging and is favorable for obtaining loosely agglomerated garnet nano-powders. However, our result shows that there is a risk of segregation and formation of rhombus Lu_2_O_3_ flakes during aging.

In fact, in the synthesis of Lu_2_O_3_ nano-powder, large-sized plates with rhombus morphologies during precipitation using AHC as the precipitant has been observed [23,24]. According to the literature, the rhombus Lu precipitate was a result of Lu(H_2_O)_x_(HCO_3_)_3_·nH_2_O formation when the pH was increased to 8. The pH value of the terminal solutions in our experiment also increased as the aging time prolonged as can be seen from Table 1. The increase is caused by the consumption of NH_4_^+^ ions forming ammonium salt [11,22]. One may argue that the formation of flakes might also be caused by the increase of pH value. In order to evaluate the effect of pH value on impurity formation, S4 and S5 were synthesized with different initial pH values.

Figure 1b shows the XRD patterns of samples of S4 and S5 calcined at 1200 °C for 3 h. No other impurity than LuAG phase could be observed in calcined S4, the terminal pH value of which is 8.35. It should be noted that S4 was only aged for 0.5 h. This result proves that the aging process was responsible for but not the sole cause of the segregation of Lu_2_O_3_. As can be seen from Figure 1b, when the initial pH value decreased to 1.20 and terminal pH to 7.32, only LuAG phase could be detected in the calcined powder that even aged for 24 h. The disappearance of Lu_2_O_3_ in samples synthesized with lower pH value implies that the formation of flakes was also affected by pH value. Low pH value is favorable for co-precipitating pure LuAG phase and relative low pH value is favorable for obtaining pure LuAG phase with the co-precipitation method. However, the pH value of terminal solution must be carefully controlled because too low of a pH value could result in incomplete precipitation of rare earth ions *etc*. [25].

In the process of synthesis of relative large amount of LuAG nano-powders with reverse-strike co-precipitation method using AHC as precipitant, dripping of large volume of salt solution into precipitant takes a long time. With the help of fierce stirring, AHC decomposition is enhanced and leads to the increase of pH value in the terminal solution. Because high terminal pH value easily causes Lu segregation during aging, it is necessary to investigate the influence of dripping rate. Figure 1c shows the corresponding XRD patterns of samples S6 and S7 calcined at 1200 °C for 3 h. S1 was free of Lu_2_O_3_ as was shown in Figure 1a. As the dripping speed decreased to 1.5 mL/min, the terminal pH value increased to 7.90 as listed in Table 1. As a result, Lu_2_O_3_ phase appeared in the calcined powder. When the dripping speed further increased to 6 ml·min^−1^, the terminal pH value decreased to 7.61 and Lu_2_O_3_ phase could not be found in its XRD pattern even when the precipitate was aged for 15 h. It was inferred that increasing dripping speed is an effective way to suppress the decomposition of AHC to obtain pure LuAG powders.

We chose 1200 °C and 3 h to calcine the dried precursors in the above study of pure LuAG nano powder formation. However, this calcining temperature might not lead to loosely agglomerated nano powders. In order to obtain LuAG nano powders suitable to sinter transparent ceramics, different temperature was applied to in calcining process.

**Figure 3:**
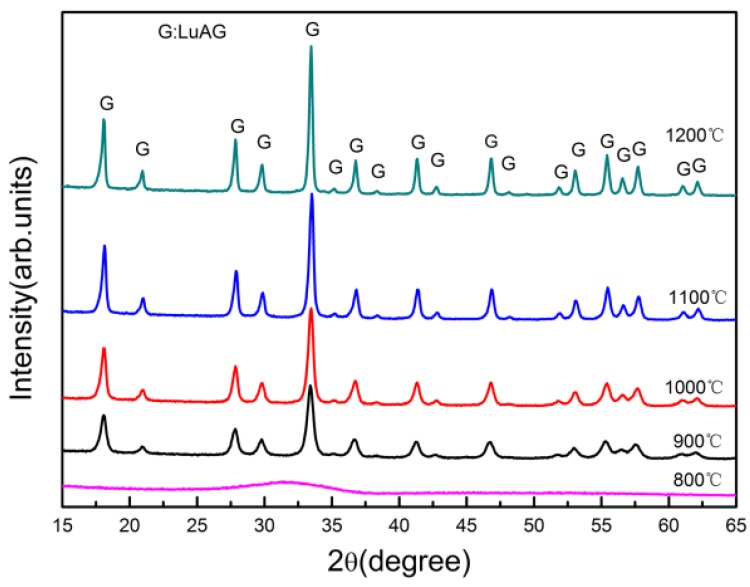
XRD patterns of S7 calcined at different temperatures for 3 h.

XRD patterns of calcined S7 at different temperatures for 3 h to form the garnet phase of LuAG powders are shown in Figure 3. The precipitate remained amorphous when the calcining temperature was 800 °C. Crystallization began at 900 °C. Further increasing temperature only resulted in increase in the intensity of the LuAG peaks, indicating crystalline growth of the LuAG powders as the temperature increased. No formation of other phases was found at any calcining temperature.

**Figure 4:**
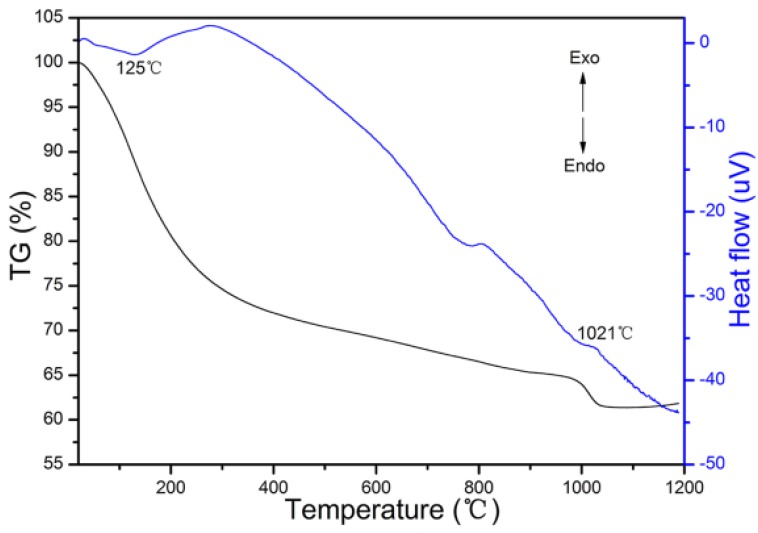
DTA-TG curves of the precursor of S7.

TG/DTA curves of the precursor of S7 are given in Figure 4. Two major peaks were identified on the DTA curve of S7. The endothermic valley that appeared at 125 °C was attributed to the evaporation of molecular water. The exothermic peak around 1021 °C in the DTA curve indicates the crystallization of LuAG phase, which is consistent with the XRD results in Figure 4 where no other phases were found. At this temperature, a weight loss of 37.67% was clearly seen, which was attributed to the final decomposition of precursor. The crystallization temperature (1021 °C) measured by TG/DTA analyzer is higher than that (900 °C) given by XRD results, which is caused by the different holding time in high temperature and the hysteresis of the TG/DTA analyzer. There was no weight loss in the TG curve above 1078 °C, which implied complete decomposition of the precursor.

**Figure 5:**
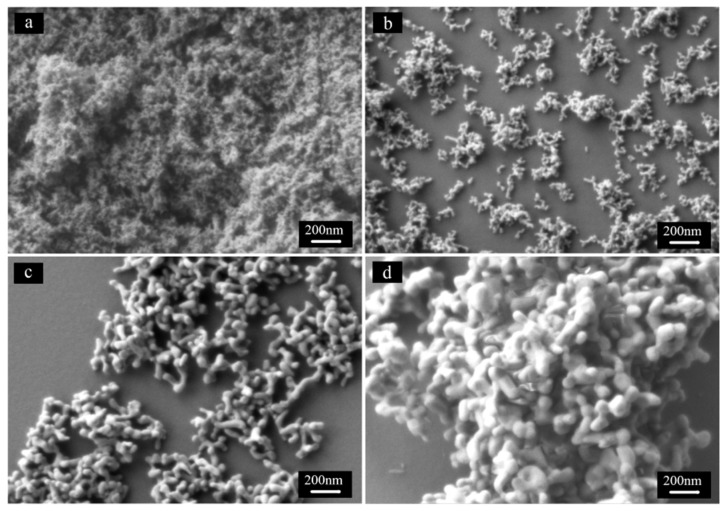
SEM images of the calcined S7 at different temperature for 3 h. (**a**) 800 °C; (**b**) 900 °C; (**c**) 1100 °C; (**d**) 1200 °C.

The morphology of the calcined S7 at different temperatures is revealed in Figure 5. The morphology of the calcined S1, S4, and S5 from 800 °C to 1200 °C is similar to that of the calcined S7 and their images were not shown here. The evolution of the particles is clearly shown in Figure 5. When calcined at 800 °C, the powder was agglomerated and no clear boundaries of particles could be distinguished. As the temperature increased to 900 °C, dispersed nano-particles were formed, accompanying the final decomposition of the precursor. As the temperature was increased to 1100 °C, well-shaped elliptical particles were developed and the average size was about 80 nm. Further increase of temperature to 1200 °C resulted in sever agglomeration and the average size of particles was about 120 nm.

**Figure 6:**
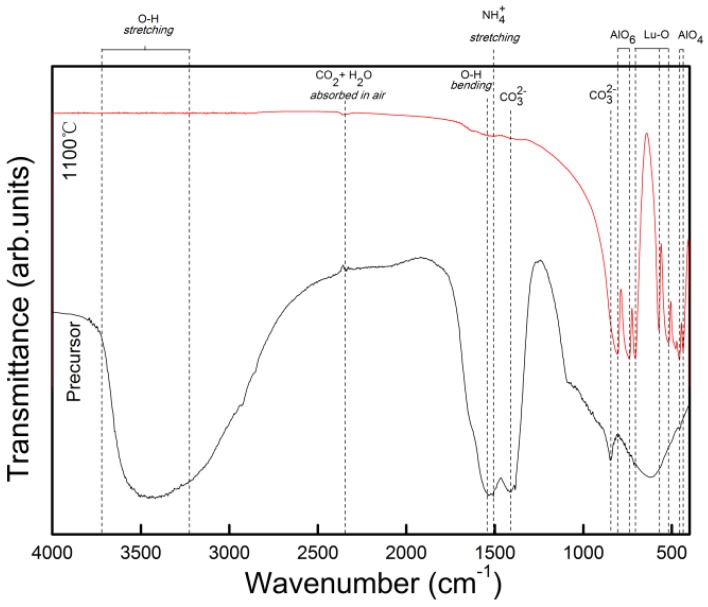
FTIR spectra of the precursor and resultant powder calcined at 1100 °C for 3 h of S7.

The FTIR spectra of the precursor of S7 and the powder calcined at 1100 °C were shown in Figure 6. The absorption valleys were assigned as shown in Figure 6. The bands at 1100 °C due to the stretching of Lu–O and Al–O bonds in the 400–800 cm^−1^ region are characteristics of LuAG, which are similar to the garnet structure of YAG [26]. No visible absorption of O–H, NH_4_^+^ or CO_3_^2−^ that all exist in the precursor was observed in the powder calcined at 1100 °C, implying complete decomposition of the precursor.

**Figure 7:**
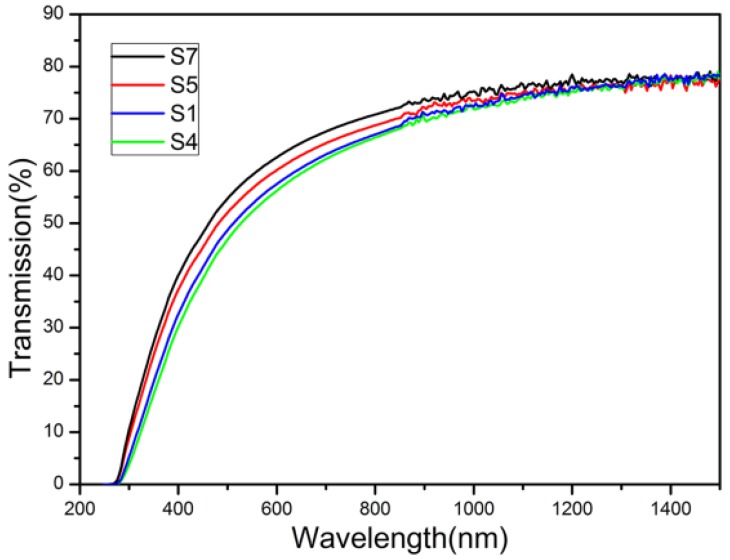
In-line transmittance of ceramics (1.5 mm in thickness) of S1, S4, S5 and S7.

Ceramics were fabricated using the nano-powders that calcined at 1100 °C from the precursors of S1–S7. However, not all of them were transparent. Ceramics fabricated from S1, S4, S5, and S7 were transparent. Their transmittance lines are shown in Figure 7. Since the others were non-transparent, their transmittance was not measured. The in-line transmittance of all the transparent ceramics is about 75% in infrared wavelength and decreases in visible wavelength range. This decrease was probably caused by Rayleigh scattering due to the pores of nano-meters. The transmittance is lower than that of LuAG ceramics fabricated by reactive solid state sintering method but similar to LuAG ceramics fabricated with LuAG nano-powders. The different transmittance in visible wavelength range for different samples indicated the difference in quality of the calcined nano-powders including particle size, dispersity, *etc*. However, this paper focuses on the segregation of rhombus Lu precipitate, its influence on the sintered ceramic and elimination of it. The parameters influencing the particle size and dispersity of the nano-powders will be studied in detail in future work.

**Figure 8:**
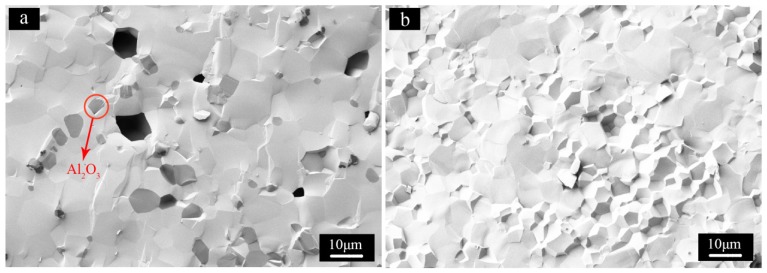
SEM micrographs of the fractured surfaces of ceramics of S3 (**a**) and S7 (**b**).

In order to investigate the influence of impurity phases on microstructures of LuAG ceramics, the fractured surfaces of ceramic samples S3 and S7 were shown in Figure 8. It is obvious that lots of pores of micrometers were located in the ceramic sintered from S3. Since calcined S3 contained Lu_2_O_3_ flakes, therefore, the pores are probably caused by the big-sized Lu_2_O_3_ flakes around which large hollows were left in the green body after slip casting and could not be totally eliminated when sintered. Dark polygonal zones were also observed in the same ceramic. EDS measurement applied in the red circle enclosing dark areas revealed the dark zones were Al_2_O_3_ phases. The appearance of Al_2_O_3_ rather than Lu_2_O_3_ is unexpected because the corresponding powder calcined at 1200 °C is rich of Lu_2_O_3_ flakes and that Al_2_O_3_ phase could not be detected by XRD measurement. The observation indicates the existence of Al_2_O_3_ in the obtained powder and clears up the doubt one may have concerning the excessive Lu_2_O_3_ in the calcined powders according to XRD measurement. The reason why Al_2_O_3_ phase was not detected by XRD may lie in the fact that the Al_2_O_3_ was amorphous or too tiny when calcined at 1200 °C for the instrument to detect until temperature increased to 1800 °C at which α-Al_2_O_3_ were formed and grew into micrometers. The disappearance of Lu_2_O_3_ phase in the ceramic might result from the reaction of Lu_2_O_3_ with Al_2_O_3_ to form intermediate phases [25] (LuAM and LuAP) or dissolution of Lu_2_O_3_ into LuAG phase during the sintering process, which cannot be distinctly observed in Figure 8a. No pores or second phase were observed in the ceramic prepared from S7. The relative density is about 99.98% of the theoretical value. The average grain size is about 10 μm. Further investigation was being made to improve quality of the sintered LuAG material.

## 3. Experimental Section

Lu_2_O_3_ (99.99%), Al(NO_3_)_3_·9H_2_O (99.99%, Tianjin Fine Chemicals, Tianjin, China) and ammonium hydrogen carbonate (Sinopharm Chemical Reagent Co., Ltd., AR, Shanghai, China) were used as starting materials. All these chemicals were used as received without further purification. The stock solution of Lu(NO_3_)_3_ was prepared by dissolving Lu_2_O_3_ into hot nitric acid and diluting with deionized water. Al(NO_3_)_3_ aqueous solution stock was made by dissolving Al(NO_3_)_3_·9H_2_O into deionized water. Cation contents in both stock solutions were assayed with the titration method. These solutions were weighed according to the stoichiometric ratio of Lu_3_Al_5_O_12_, diluted to achieve a Lu^3+^ content of 0.1 mol·L^−1^ and mixed homogeneously. The pH value of the mixed solution was adjusted with HNO_3_. (NH_4_)_2_SO_4_ (Sinopharm Chemical Reagent Co. Ltd., Shanghai, China, analytical purity) was added into the mixed solutions as dispersant. Precipitant solution with a concentration of 2 mol·L^−1^ and pH = 8.50 was obtained by dissolving NH_4_HCO_3_ in deionized water. The precursor precipitate was made at 17 °C by dripping 1200 mL of the mixed nitrate solutions into 800 ml of the precipitant at a speed of 1.5–6 mL·min^−1^ under fierce agitation. After titration, the suspensions were aged for 1–24 h without agitation subsequently centrifuged and washed six times, of which the former three times were with deionized water, latter three times with ethanol. Titration conditions such as aging time, dripping speed, and related pH values of solutions are listed in Table 1. The precipitates were dried at 70 °C for 24 h. The dried cakes were crushed with a corundum pestle and mortar, and sieved through a 200-mesh screen. The sieved precursor powders were calcined at different temperatures for 3 h to form the garnet phase of LuAG powders.

The LuAG transparent ceramics were fabricated by slip casting and vacuum sintering method. TEOS (0.5 wt %, 99.99%, Aladdin, Shanghai, China) was added to the calcined LuAG powders as sintering aids. The mixture was blended with the high-purity ZrO_2_ balls for 3 h in distilled water. To obtain stable slurry, 1.5 wt % of ammonium polymethacrylate was added to the above mixture before milling. The slurry was then cast into plaster molds and dried in air to form green bodies. After drying, the green bodies were calcined at 800 °C for 3 h to remove water and organics, followed by sintering in vacuum below 1.0 × 10^−2^ pa at 1800 °C for 10 h and then annealed at 1450 °C for 10 h in the air.

The pH value of solutions before precipitation and after aging was monitored using a digital pH meter with an accuracy of 0.01 (SevenEasy S20, Mettler Toledo, Shanghai, China). The phase properties and elemental chemical composition were characterized using X-ray diffraction (XRD, Rigaku Co., Tokyo, Japan) and Energy Dispersive Spectrometer (EDS, Hitachi, Tokyo, Japan), respectively. Morphologies of the LuAG powders and of the fresh fracture surfaces of LuAG ceramics were examined using scanning electron microscopy (SEM, JEOL, Tokyo, Japan). Thermal behaviors of the LuAG precursors were studied using thermogravimetry analysis and differential thermal analysis (TG/DTA7300, EXSTAR Series, JEOL, Tokyo, Japan) from room temperature to 1200 °C at a heating rate of 10 °C·min^−1^ in air, and the alpha alumina was used as a reference. The compositions of the precipitates were investigated by the Fourier transform infrared (FTIR) spectrometer (Thermo Nicolet Nexus, Youngstown, OH, USA) with KBr pellet. The in-line transmittance of mirror-polished samples was measured by a spectrometer (λ750, Perkin Elemer, Waltham, CT, USA). The density of the sintered ceramics was determined by the Archimedes method.

## 4. Conclusions

To achieve high transmittance, fine grain structure, and full density in LuAG ceramics is still a challenge. Fine and pure powders without or only with slight agglomeration is necessary for high quality of sintered LuAG ceramics. The significance of this work is providing basic parameters to synthesize pure phased and well dispersed LuAG nano-powder. By accelerating the dripping speed or decreasing the initial pH value to a reasonable degree, the segregation of rhombus Lu precipitate and fine Al precipitate was eliminated and single phase LuAG powders were obtained. Through LuAG precursors were calcined at 1100 °C for 3 h to obtain powder with slight agglomeration. Finally, transparent LuAG ceramics were fabricated with the synthesized LuAG nano powders and the transmission is about 75.5% at the wavelength of 1064 nm.

Results highlights and supplements:

1 The Combined effect of pH and time. Higher pH value of terminal solution, longer titration time, and longer aging time resulted in the appearance of rhombus Lu precipitates.
2 The evolution of impurities. From the morphology of flake-like Lu_2_O_3_, it is obvious that rhombus Lu precipitate transformed into Lu_2_O_3_ upon calcinations at 1200 °C for 3 h. It is not easy for big sized rhombus Lu precipitate to react with fine Al precipitate to transform into LuAG phase because of long diffusion distance. As a consequence, there were two kind of impurity phases in the LuAG powder of S3, one was flake-like Lu_2_O_3_, the other was fine Al_2_O_3_.
3 The dispersity of powder. It is an easy way to obtain well dispersed LuAG powder by calcining at different temperatures. Higher calcination temperature resulted in drastic increase in crystallite size and severed agglomeration with decreasing sinterability. While the powders produced at lower temperature, smaller crystallite size, and high sintering activity caused to abnormal grain growth and intragranular porosity in the sintering process. The excellent dispersion and sintering properties of powder need to further change synthesis conditions, including species and dosage of dispersant, co-precipitation temperature and so on.
4 The transmittance of ceramics. It is true that our samples were not as transparent as solid-state reactive sintered LuAG laser ceramics. However, there is a strong segregation of rare earth ions at grain boundaries in solid-state reactive sintered ceramics, which affects the performance in laser applications. Using synthesized nano-powders via AHC co-precipitation could solve this problem. Besides, the transmittance of our samples is superior or comparable to LuAG ceramics using the synthesized LuAG nano powders via AHC co-precipitation method recently reported results. Decreasing the agglomeration of the synthesized LuAG nano-powders, refining forming, and optimizing the sintering process can improve the transmittance of transparent LuAG ceramics. Research on these subjects are underway in our laboratory.
